# Nailfold Videocapillaroscopic Alterations as Markers of Microangiopathy in COVID-19 Patients

**DOI:** 10.3390/jcm12113727

**Published:** 2023-05-28

**Authors:** Roberta Gualtierotti, Sharon E. Fox, Fernanda Da Silva Lameira, Andrea Giachi, Luca Valenti, Maria Orietta Borghi, Pier Luigi Meroni, Massimo Cugno, Flora Peyvandi, on behalf of the CAPCOVID Study Group

**Affiliations:** 1 S.C. Medicina—Emostasi e Trombosi, Centro Emofilia e Trombosi Angelo Bianchi Bonomi, Fondazione IRCCS Ca’ Granda, Ospedale Maggiore Policlinico di Milano, 20122 Milan, Italy; 2 Department of Pathophysiology and Transplantation, Università Degli Studi di Milano, 20122 Milan, Italy; 3 Department of Pathology, Louisiana State University Health Sciences Center, New Orleans, LA 70802, USA; 4 Pathology and Laboratory Medicine Service, Southeast Louisiana Veterans Healthcare System, New Orleans, LA 70119, USA; 5 Omic Science and Precision Medicine Laboratory, Biological Resource Center, Transfusion Medicine, Fondazione IRCCS Ca’ Granda, Ospedale Maggiore Policlinico, 20122 Milan, Italy; 6 Department of Clinical Sciences and Community Health, Università Degli Studi di Milano, 20122 Milan, Italy; 7 Immunorheumatology Research Laboratory, IRCCS Istituto Auxologico Italiano, 20095 Milan, Italy

**Keywords:** nailfold videocapillaroscopy, COVID-19, endothelial perturbation, Von Willebrand factor, hemosiderin

## Abstract

Nailfold videocapillaroscopic alterations have been described in COVID-19, but their correlations with biomarkers of inflammation, coagulation and endothelial perturbation are still unclear, and no information is available on nailfold histopathology. Nailfold videocapillaroscopy was performed on fifteen patients with COVID-19 in Milan, Italy and the signs of microangiopathy were correlated with plasma biomarkers of inflammation (C reactive protein [CRP], ferritin), coagulation (D-dimer, fibrinogen), endothelial perturbation (Von Willebrand factor [VWF]) and angiogenesis (vascular endothelial growth factor [VEGF]) along with genetic drivers of COVID-19 susceptibility. Histopathological analysis of autoptic nailfold excisions was performed on fifteen patients who died for COVID-19 in New Orleans, United States. All COVID-19 patients studied with videocapillaroscopy showed alterations rarely seen in healthy individuals consistent with microangiopathy, such as hemosiderin deposits (sign of microthrombosis and microhemorrhages) and enlarged loops (sign of endotheliopathy). The number of hemosiderin deposits correlated both with ferritin and CRP levels (r = 0.67, *p* = 0.008 for both) and the number of enlarged loops significantly correlated with the levels of VWF (r = 0.67, *p* = 0.006). Ferritin levels were higher in non-O groups, determined by the rs657152 C > A cluster, (median 619, min–max 551–3266 mg/dL) than in the O group (373, 44–581 mg/dL, *p* = 0.006). Nailfold histology revealed microvascular damage, i.e., mild perivascular lymphocyte and macrophage infiltration and microvascular ectasia in the dermal vessels of all cases, and microthrombi within vessels in five cases. Alterations in nailfold videocapillaroscopy and elevated biomarkers of endothelial perturbation that match histopathologic findings open new perspectives in the possibility of non-invasively demonstrating microangiopathy in COVID-19.

## 1. Introduction

Increasing evidence supports microvascular involvement in the pathogenesis of lung and multi-organ dysfunctions in COVID-19 [1,2]. An exaggerated response to SARS-CoV-2 is believed to be the main event underlying microvascular perfusion impairment through inflammation, complement and coagulation activation, and endothelial dysfunction [2,3].

Nailfold videocapillaroscopy is a non-invasive, safe and simple technique that allows in vivo assessment of the morphological and functional aspects of microcirculation [4]. Nailfold videocapillaroscopy has been largely used not only for investigating peripheral microangiopathy, but also as a sort of “window” to systemic microvascular dysfunction. Its main applications are in detecting the transition from primary to secondary Raynaud’s phenomenon [5] and in connective tissue diseases such as systemic sclerosis [6] and dermatomyositis [7]. Recently, its value has emerged in other fields of internal medicine characterized by endothelial dysfunction [8]. The most relevant capillaroscopic alterations include enlargement of capillaries (enlarged or giant loops), avascular areas and hemosiderin deposits due to microhemorrhages or microthrombosis. Overall, these alterations are conventionally referred to as “scleroderma pattern” [9,10]. Microhemorrhage, which is the result of endothelial injury and blood extravasation, appears as a pearl-necklace hemosiderin deposit. Microthrombosis appears as hemosiderin deposits that trace the shape of the truncated capillary loop as a result of the arrest of the blood column within the capillary [11]. Although the main application of nailfold videocapillaroscopy is within the connective tissue diseases of the scleroderma spectrum, it has been employed in non-rheumatic diseases with a microvascular involvement such as diabetes mellitus and essential hypertension [12]. During COVID-19 pandemic, the nailfold videocapillaroscopy has been performed for the evaluation of microcirculation in patients with different degrees of disease severity [13,14,15,16]. Natalello et al., who evaluated nailfold videocapillaroscopy in COVID-19 adult patients, reported a higher prevalence of hemosiderin deposits in acutely ill patients, as well as a higher number of enlarged and meandering loops with a lower capillary density in patients who recovered. All these findings have been considered consistent with prothrombotic alterations of the microcirculation. However, none of the findings correlated with markers of inflammation (C-reactive protein [CRP]) and coagulation (D-dimer), both during the acute phase and during a two-month follow-up [16]. Similarly, Rosei et al. demonstrated the presence of microhemorrhages and microthrombosis in acute patients and a reduction of capillary density three months after the acute infection. Capillary density inversely correlated with maximum CRP and ferritin levels and with minimum lymphocyte count during the acute phase of the disease [13]. Sulli et al. showed that the reduction in the absolute capillary number in adult patients affected by COVID-19 persisted several months from recovery [14]. In a pediatric population affected by COVID-19, Çakmak et al. reported a higher frequency of microhemorrhages and a correlation between capillary abnormalities and both CRP and D-dimer levels [15]. Nailfold videocapillaroscopy also revealed signs of nailfold microangiopathy [17] in a subgroup of patients with dermatological manifestations induced by COVID-19 resembling chilblain, i.e., erythematous to violaceous papules and plaques of the acral portion of fingers and toes [18].

Although previous studies have described nailfold videocapillaroscopic changes suggestive of microvascular involvement in patients with COVID-19, the link of these alterations with actual microvascular damage has not been clarified. With this as background, the aim of our study was to evaluate in COVID-19 patients the in vivo alterations of nailfold videocapillaroscopy (such as number of capillaries, enlargement of loops and presence of hemosiderin deposits) [13,14,15,16,19], and of inflammation, coagulation and endothelial biomarkers, as well as the post-mortem histopathological counterpart in nailfold excision samples obtained at autopsy from patients dead for COVID-19.

## 2. Materials and Methods

### 2.1. Patients

The present observational study has been conducted on two cohorts of patients. The first cohort included fifteen consecutive patients admitted to the Internal Medicine unit of Fondazione IRCCS Ca’ Granda Ospedale Maggiore Policlinico, Milan, Italy from April 2020 to May 2020. Inclusion criteria were: confirmed diagnosis of COVID-19 and age > 18 years; exclusion criteria were: pre-existing Raynaud’s phenomenon or history of connective tissue disease. Blood sample collection and nailfold videocapillaroscopy were performed within 72 h from hospitalization. Patient outcomes were assessed at discharge from the hospital and up to three months later. As normal controls for nailfold videocapillaroscopy, fifteen healthy subjects were matched with patients with COVID-19 for sex, age and main comorbidities (Table 1). All subjects provided written informed consent.

The study was approved by the Ethics Committee of Fondazione IRCCS Ca’ Granda Ospedale Maggiore Policlinico in Milan (No. 360_2020) and was carried out in conformity with the 2013 revision of the Declaration of Helsinki and the code of Good Clinical Practice.

The second cohort, from New Orleans, LA, USA, included 15 patients with death from COVID-19 and five controls with death from non-COVID-19 pneumonia complications. Nailfold excisions were performed during autopsy and analyzed by histology during the same period (i.e., April–May 2020). Consent for autopsy without restriction was given by the next of kin, and the studies were determined to be exempt from oversight by the Institutional Review Board of the Southeast Louisiana Veterans Healthcare System.

### 2.2. Nailfold Videocapillaroscopy

Images were acquired using a digital videocapillaroscopy system with a dedicated software for image analysis (5MP USB 3.0, INSPECTIS© CAP Pro, Kista, Sweden). The probe is a modified type of immersion microscope with a 200× magnification high-resolution lens and built-in illumination. Videocapillaroscopy is performed at the nailfold, where capillaries are arranged with the longitudinal axis parallel to the skin surface, so that they can be examined along their entire length [7].

The procedure was conducted by a physician with experience in videocapillaroscopy (RG). Following the application of maple oil, all fingers except the thumb were examined bilaterally as previously described [10]. The first finger was not examined according to the recommendations due to the thickened skin layer that does not allow optimal evaluation of the capillaries [20]. For each image, the following parameters were studied: the total number of capillaries in 1 mm-width in the distal row, the loop diameter (external, internal and apical diameters), the number of capillaries with different morphological aspects (i.e., hairpin shaped loops, loops with one cross or with two or more intersections, meandering and bushy loops) [9], and the presence of microthrombosis and microhemorrhages [21].

We defined abnormalities as follows: enlarged loop as a capillary with increased diameter (homogeneous or irregular) >20 μm and <50 μm [18]; giant capillary as an homogeneously enlarged loop with an apical diameter ≥ 50 μm; hemosiderin deposits as dark masses due to microhemorrhage (single or multiple round-shaped hemosiderin deposit at variable distance from the distal row, as the result of blood extravasation) or microthrombosis (hemosiderin deposit that traces the shape of a truncated capillary loop, as the result of blood column arrest within the capillary) [21]; low capillary density as the reduction of the capillary number below the average of 7 capillaries per mm [10]; bushy capillaries as capillaries with ramifications often originated from a single normal-sized capillary [22]; loops with one cross or with two or more intersections as limbs crossing one or two times, respectively [23]; meandering loop as a capillary with limbs crossing more than two times [24].

### 2.3. Laboratory Tests

Routine laboratory analyses were performed during hospitalization. In order to exclude an autoimmune pathogenesis, we tested patients for a panel of autoantibodies including: antinuclear antibody (ANA) by indirect immunofluorescence on HEp2 cells, considering positive those samples with a dilution ≥ 1:80 [25]; anti-extractable nuclear antibodies (anti-ENA), detected by DotBlot EUROLINE Systemic Sclerosis and Myositis profile IgG (EUROIMMUN AG, Lubeck, Germany) [26]; anti-double strand (ds) DNA by fluorimetric analysis (Elia, Thermo Fisher Scientific, Waltham, MA, USA) [27]. ABO groups were determined by genotyping in our cohort due to the known influence on Von Willebrand factor (VWF) plasma levels as previously described [28,29]. In addition, the ABO blood group and genetic variations associated with COVID-19 susceptibility, namely chromosome 3 cluster rs11385942 G > GA, the main genetic determinant of COVID-19 severity, ABO rs657152 C > A a main determinant of ABO blood group and the main genetic determinant of COVID-19 infection susceptibility, and FUT2 rs601338 G > A encoding for the ABO secretor phenotype and a modulator of COVID-19 severity, were evaluated as previously described [3,30,31,32,33]. Briefly, DNA was extracted from peripheral blood collected at the time of enrollment by the QIASymphony (Qiagen, Milan, Italy). Genotyping was performed by Illumina Global Screening Array (GSA)-24 v3.0 plus Multidisease Array (Illumina, San Diego, CA, USA), which contains 712,189 variants before quality control. To maximize genetic coverage, we performed single-nucleotide polymorphism (SNP) imputation on genome build GRCh38 using the Michigan Imputation Server and 194,512 haplotypes generated by the Trans-Omics for Precision Medicine (TOPMed) program (freeze 5). ABO blood group was imputed based on rs657152 C > A (which were directly type by the Illumina_GSA v3.0 chip).

Levels of the proangiogenic vascular endothelial growth factor (VEGF) were measured using the Human VEGF Quantikine ELISA Kit (R&D Systems inc., Minneapolis, MN, USA), while VWF antigen was tested as a marker of endothelial perturbation with an ELISA method previously described [34].

### 2.4. Histopathology

Nailfold skin samples were excised from the second or third finger at the time of autopsy, formalin-fixed and paraffin-embedded. Subsequently, they were examined by an experienced dermatopathologist on both hematoxylin and eosin and iron stains. If macrophages were observed histologically, CD68 immunohistochemistry was performed for confirmation [35].

### 2.5. Statistical Analysis

Categorical variables were reported as number and percentage, and continuous variables as median and range (min–max). We used the non-parametric test of Spearman’s Rho to evaluate the correlations between videocapillaroscopic alterations and clinical and laboratory parameters as well as biomarkers of inflammation, coagulation and endothelial dysfunction. Differences between COVID-19 patients vs. healthy subjects as well as within COVID-19 patients with or without ANA positivity, and with O or non-O blood groups were evaluated by the non-parametric test of Mann–Whitney U test. Differences in percentage of videocapillaroscopic alterations were evaluated by means of chi-square test. Statistical significance was defined as a *p*-value < 0.05.

The sample size of patients included in the present study allowed to obtain a statistical power of 80% with an alpha error of 0.05, based on the study by Ingegnoli et al. [23] considering a frequency of 2% of hemosiderin deposits in healthy subjects. Data were analyzed using IBM SPSS Statistics v27.0 (IBM Corp. Released 2020. IBM SPSS Statistics for Windows, Version 27.0. Armonk, NY, USA: IBM Corp.).

## 3. Results

### 3.1. Clinical, Laboratory and Nailfold Videocapillaroscopic Findings of COVID-19 Patients in the Milan Cohort

Demographic and clinical characteristics of the Milan cohort are reported in Table 1. Hematological and biochemical parameters at evaluation are reported in Table 2.

Plasma levels of fibrinogen were inversely correlated with the time from symptom onset (r = −0.59, *p* = 0.02). Median levels and up to 75% of values of fibrinogen, D-dimer, ferritin and CRP were higher than the normal range (Table 2). Similarly, median levels and up to 75% of values of VWF were above the upper limit of the normal range (Table 3).

The other results of the second-level laboratory and genetic analyses are also reported in Table 3. In particular, ferritin levels were significantly higher in patients with the rs657152 C > A cluster, associated with a worse prognosis and determining non-O groups (median 619, min–max 551–3266 mg/dL) than in patients without the cluster, i.e., with the O group (median 373, min–max 44–581 mg/dL, *p* = 0.006). The genetic analysis of other susceptibility loci of the patients revealed no difference in nailfold videocapillaroscopic nor in biochemical parameters in patients with the rs11385942 G > GA and the rs601338 G > A variants. Median VEGF levels were normal and three of the patients had a positive ANA test, one with cytoplasmic pattern (Anti-Cell [AC]-16), non-specifically associated with systemic autoimmune rheumatic diseases, and the other two with a fine-speckled (AC-4) and a nucleolar pattern (AC-8). However, none of these patients had signs and symptoms of a connective tissue disease and anti-ENA and anti-dsDNA antibodies were negative in all three patients. In addition, no difference in nailfold videocapillaroscopy characteristics nor in biochemical parameters were found compared to the ANA-negative patients.

**Figure 1:**
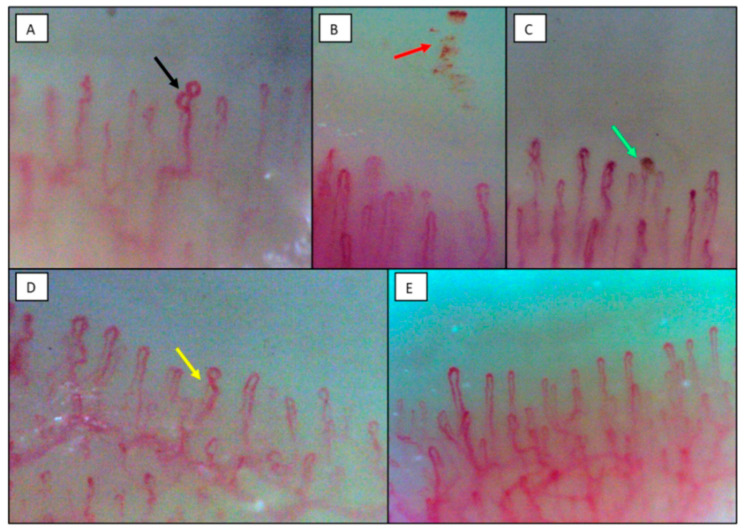
Capillaroscopic findings in patients with mild COVID-19. (**A**), enlarged loops (black arrow); (**B**), pearl necklace appearance of microhemorrhages (red arrow); (**C**), microthrombosis (green arrow); (**D**), one-loop cross (yellow arrow); (**E**), normal.

All 15 enrolled patients showed alterations in the capillaroscopic pattern (Table 4, Figure 1). The most relevant finding was the presence of at least one hemosiderin deposit in 93.3% of patients, which was significantly different from healthy controls (26.7%, *p* < 0.001). The median number of these deposits was 4 (range 0–27) vs. 0 (0–1) in healthy subjects (*p* < 0.001) and the median number of fingers involved was 2 (range 0–8). In particular, at least one microhemorrhage was found in 14 (93.3%) patients, whereas hemosiderin deposits with the aspect of microthrombosis were found in four patients (26.7%). More than one enlarged loop was present in 66.7% of patients, compared with 12.5% in healthy controls (*p* = 0.002) and as a consequence, internal loop distance was significantly reduced (*p* = 0.02). Other significantly different findings compared with healthy subjects were loop length, which was increased (*p* = 0.01), a higher number of individuals with at least one loop with two or more intersections (100% vs. 46.7%, *p* = 0.002), with at least one meandering capillary (66.7% vs. 6.7%, *p* < 0.001) and with at least one bushy capillary (73.3% vs. 6.7%, *p* < 0.001). The number of capillaries per mm was not significantly different from healthy controls. The comparison between patients and healthy controls for each videocapillaroscopic alteration is reported in Table 4.

The number of hemosiderin deposits was significantly correlated with ferritin and CRP plasma levels (r = 0.67, *p* = 0.008 for both). A direct correlation was also evident between the number of microhemorrhages and ferritin (r = 0.65, *p* = 0.001) or CRP plasma levels (r = 0.69, *p* = 0.005). VWF levels were significantly correlated with the number of enlarged loops (r = 0.67, *p* = 0.006). Capillaroscopic alterations did not correlate with age, metabolic parameters, or with the clinical outcome at discharge and at a 3-month follow-up. In particular, all the patients survived and recovered irrespective of the acute-phase alterations.

### 3.2. Clinical and Histopathological Findings in Patients Died of COVID-19 in the New Orleans Cohort

**Figure 2:**
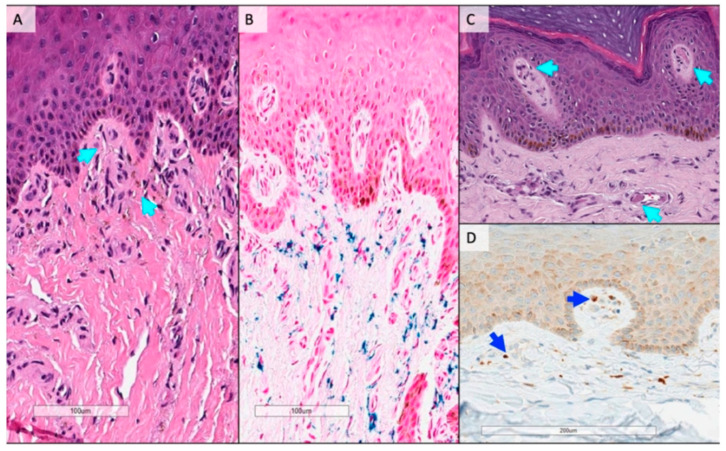
Histopathological images of nailfold skin from patients with death due to COVID-19. (**A**), dilated capillary loops in the papillary dermis with a mild chronic inflammatory infiltrate and hemosiderin deposition (teal arrows); (**B**), iron stain confirming hemosiderin deposition in the papillary and superficial dermis with occasional hemosiderin-laden macrophages (blue); (**C**), dilated blood vessels in the papillary and superficial dermis with blood clot present (teal arrows); (**D**), a CD68 immunostaining confirming the presence of hemosiderin-laden macrophages in the same distribution (blue arrows).

Demographics and clinical data of patients from the autopsy cohort is reported in Table 5. In patients with death due to COVID-19 no macroscopic alteration was evident with the exception of rare darkening of the skin near the nailfold. In the dermal vessels of all COVID-19 cases, histologic examination revealed evidence of microvascular damage, including a mild, superficial dermal perivascular lymphocytic infiltrate, with neutrophils at times lining the capillary endothelium, as well as apparent microvascular ectasia (Figure 2, panel A). Red blood cell extravasation, hemosiderin deposits identified on iron stains, associated macrophages and dermal edema were observed in COVID-19 patients (Figure 2, panel B). There was no definitive evidence of a true vasculitis. Five cases demonstrated microthrombi within vessels (Figure 2, panel C). The macrophage-specific CD68 immunostaining confirmed the presence of hemosiderin-laden macrophages in the same distribution (Figure 2, panel D). Five patients dead for non-COVID-19 pneumonia complications of the same age range and sex ratio of COVID-19 patients were included as controls. In these subjects, the histology of nailfold capillaries showed no evidence of microvascular damage, no hemosiderin deposits, nor microthrombi within the vessels.

## 4. Discussion

In hospitalized patients with COVID-19 during the first wave of pandemic, in vivo alterations in nailfold videocapillaroscopy correlated with biomarkers of inflammation, coagulation and endothelial dysfunction, and were consistent with the histopathological findings of microangiopathy at nailfold skin excisions in a cohort of patients who died from COVID-19. In particular, the videocapillaroscopic alterations of loop enlargement, microhemorrhages and microthrombi correspond to the histopathological findings of capillary ectasia, hemosiderin-laden macrophages and the intracapillary microthrombi, respectively.

Nailfold videocapillaroscopy showed pathological alterations in almost all COVID-19 patients in the form of enlarged loops, a sign of endothelial distress, hemosiderin deposits, a sign of microhemorrhage and, to a lesser extent, microthrombosis. In addition, we found significantly reduced internal loop distance, which is a consequence of loop enlargement. The presence of meandering and bushy capillaries has been previously reported in the acute phase of COVID-19 by other authors and related to angiogenesis [15,19]. The number of capillaries per mm was not significantly different from healthy controls, in agreement with reported findings during the acute SARS-CoV2 infection [19]. The abnormality of other videocapillaroscopic parameters such as increased loop length and loops with two or more intersections are considered non-specific abnormalities [10].

These alterations are consistent with the observation that the majority of our COVID-19 patients had high plasma levels of VWF, a reliable marker of endothelial involvement [36,37,38,39], and these levels were significantly correlated with the number of enlarged loops. Indeed, in the general population non-O group individuals have higher VWF levels compared to group-O individuals, whereas in our patients VWF plasma levels were increased irrespective of the blood group, as observed in the course of inflammation or endothelial stimulation [40]. ABO groups were not correlated with any other tested parameter, with the exception of ferritin that was higher in non-O group patients than in O-group patients. As expected, the rs657152 C > A SNP at the ABO genetic locus, which was used to infer the ABO blood group showed overlapping results [30,31]. The other two investigated susceptibility risk variants, chromosome 3 cluster rs11385942 G > GA, at locus 3p21.31 and chromosome 19 rs601338 G > A FUT2 [3,32,41], had no correlation with the tested parameters.

Previous studies have explored the different nailfold videocapillaroscopic findings and in some cases laboratory tests in COVID-19 patients during the acute phase, from 48 h to 7 days from admission [13,16]. However, some differences must be highlighted. Natalello et al. identified specific nailfold videocapillaroscopy alterations in acute and recovered mild COVID-19 patients in agreement with our findings [16]. The study from Rosei et al. mainly found a significant reduction of capillary density of COVID-19 patients after three months from the acute infection and demonstrated how the microthrombosis or microhemorrhages that appear in the acute phase of the disease tend to disappear at follow-up [13]. Accordingly, the only significantly altered parameter in the nailfold bed of adult COVID-19 survivors found by Sulli et al. was a reduction in the absolute number of capillaries after a follow-up of 60 days up to 317 days [14]. Finally, in a pediatric population affected by COVID-19, Çakmak et al. reported a higher frequency of microhemorrhages. In addition, a correlation between capillary abnormalities and both CRP and D-dimer levels was demonstrated [15].

The signs of microangiopathy observed at nailfold videocapillaroscopy are consistent with the post-mortem findings at nailfold excisions. In particular, the images of the enlarged loops at videocapillaroscopy correspond to the histological finding of dilated capillaries or capillary ectasia in the papillary and superficial dermis; the microhemorrhages observed at videocapillaroscopy correspond to the hemosiderin-laden macrophages and surrounding iron staining; finally, the microthrombi observed at videocapillaroscopy are consistent with the histopathological finding of intracapillary microthrombi. Overall, dermal infiltrate, microvascular ectasia, hemosiderin deposits and microthrombi are consistent with microangiopathy and are not observed in critical illness not due to COVID-19.

One of the limitations of this study is the small number of patients enrolled, due to the challenge of equipment use in the COVID-19 ward, and sanitization of a sophisticated instrument such as the capillaroscopic system in the course of the first wave of pandemic. However, the sample size did reach an adequate power and alpha error. The correlations shown are based on a non-parametric measure of rank correlation (Spearman’s rho) and no multiple variable adjustments have been made due to the small sample size. A further limitation was the fact that the nailfold histopathological analysis was not performed in samples from the same patients in which the nailfold videocapillaroscopy was conducted. This was due to the fact that the nailfold biopsy is not a current practice in our patients and particularly in COVID-19, for which in our hospital an adequate biosafety equipment for histopathology was not available. In contrast, the New Orleans Center has demonstrated a consolidated expertise on histopathology in COVID-19 with adequate biosafety equipment [42].

One strength of our study is the concurrent measurement of inflammation, coagulation and endothelial perturbation serological biomarkers. Another strength is the availability of unique histological findings that mirrored the in vivo capillaroscopic features. In addition, histopathology with immunohistochemistry was able to confirm the presence of CD68+ macrophages, which have been associated with damage to other organs in cases of moderate to severe COVID-19 [35]. The non-diagnostic serological profiling of the patients ruled out the possibility of an underlying systemic autoimmune disorder.

These data are particularly relevant because they were collected during the first wave of COVID-19 pandemic, when the high risk of infection seriously limited the use of state-of-the-art instruments for the diagnosis and monitoring of endothelial dysfunction in the ward. In addition, we had the opportunity to study patients who did not undergo any specific treatment for the disease, as little was known at the time about the pathogenesis of COVID-19, and the main therapeutic approach was supportive care.

Overall, these findings support the view that in COVID-19 patients, endothelial perturbation may lead to peripheral microvascular dysfunction, which can be disclosed by nailfold videocapillaroscopy. A similar scenario has been found in systemic sclerosis and in other conditions characterized by endothelial dysfunction, where the extent of microangiopathy observed at nailfold videocapillaroscopy has been demonstrated to correlate with internal organ involvement [5,6,7,8].

## 5. Conclusions

The observation by nailfold videocapillaroscopy of microangiopathy in COVID-19 is supported by elevated biomarkers of endothelial perturbation and confirmed by the corresponding nailfold histopathological findings. These results open new perspectives for the in vivo assessment of the endothelial perturbation with a non-invasive and reliable tool, not only during the acute phase of COVID-19, but also during the follow-up of its long-term sequelae.

## Figures and Tables

**Table 1:** Demographic and clinical characteristics of the Milan cohort and matching healthy controls.

	COVID-19 Patients	Healthy Controls	Significance
AGE (years)			
Median (range)	56.0 (40.0–84.0)	52 (39.0–82.0)	n.s.
SEX			
M	10 (66.7%)	10 (66.7%)	
F	5 (33.3%)	5 (33.3%)	n.s.
BMI (kg/m^2^)		n.a.	
Median (range)	28.4 (21.1–37.5)		
Missing	6 (40.0%)		
BLOOD PRESSURE AT ADMISSION (mmHg)			
Systolic blood pressure, median (range)	125 (105–145)	n.a.	
Diastolic blood pressure, median (range)	80 (60–90)		
TIME FROM ONSET (days)			
Median (range)	3.7 (1.6–6.6)	n.a.	
MAIN COMORBIDITIES			
Arterial Hypertension	6 (40%)	5 (33.3%)	n.s.
Betablockers	3 (20%)	2 (13.3%)	n.s.
Diuretics	3 (20%)	1 (6.7%)	n.s.
ACE inhibitors	1 (6.7%)	2 (13.3%)	n.s.
ARB	4 (26.7%)	1 (6.7%)	n.s.
Dyslipidemia	4 (26.7%)	3 (30%)	n.s.
Statin	3 (20%)	2 (13.3%)	n.s.
Diabetes	2 (13.3%)	1 (6.7%)	n.s.
OXYGEN THERAPY			
Total	12 (80%)	n.a.	
Non-invasive ventilation	4 (26.7%)		
Invasive ventilation	0		
TREATMENT FOR COVID-19		n.a.	
Steroids	4 (26.7%)		
LMWH	14 (93.3%)		
Remdesivir	3 (20%)		
Hydroxychloroquine	9 (60%)		
Biological drugs (tocilizumab)	1 (6.7%)		

ACE angiotensin converting enzyme; ARB angiotensin receptor blocker; BMI body mass index; LMWH low-molecular weight heparin; n.a. not available; n.s. not significant; Non-invasive ventilation Continuous positive air pressure or bilevel positive air pressure.

**Table 2:** Hematological and biochemical parameters of the Milan cohort.

Parameter	Median (Range)	Reference Range
WBC/mm^3^	6900 (3170–13,780)	(4800–10,800)
Neutrophils/mm^3^	4290 (1800–10,410)	(1500–6500)
Lymphocytes/mm^3^	1510 (650–2760)	(1200–3400)
Hemoglobin, g/dL	12.2 (9.6–15.5)	(13.5–17.5)
MCV, fL	84.1 (76.4–94.5)	(80–94)
Platelets, ×10^3^/mm^3^	344 (175–529)	(130–400)
MPV, fL	10.0 (8.4–11.3)	(9.5–13.1)
FBG, mg/dL	419 (252–894)	(165–350)
D-dimer, ng/mL	991 (437–2128)	(<500)
Ferritin, mg/dL	587 (44–3266)	(30–400)
Creatinine, mg/dL	0.85 (0.44–1.23)	(0.72–1.18)
CRP, mg/dL	1.03 (0.06–18.31)	(<0.5)
ALT, IU	43 (18–109)	(9–59)
LDH, IU	215 (168–333)	(135–225)
Glucose, mg/dL	94 (65–311)	(70–110)
Triglycerides, mg/dL	139 (78–306)	(<150)
Total cholesterol, mg/dL	151 (110–184)	(<190)

ALT alanine transaminase; CRP C-reactive protein; FBG fibrinogen; IU international unit; LDH lactate dehydrogenase; MCV mean corpuscular volume; MPV mean platelet volume; WBC white blood count.

**Table 3:** Second-level laboratory and genetic assessment of the Milan cohort.

Parameter	Median (Range)	Reference Range
VEGF, pg/mL, median (range)	156 (25–310)	(62–707)
VWF, %, median (range)	198 (48–704)	(50–130)
Blood type, n (%)		
A	6 (40.0%)	
AB	2 (13.3%)	
B	1 (6.7%)	
O	6 (40.0%)	
Chromosome 3 cluster rs11385942 G > GA, n (%)	3 (20%)	
Chromosome 9 cluster rs657152 C > A, n (%)	9 (60%)	
Chromosome 19 cluster rs601338 G > A, n (%)	6 (40%)	
ANA positivity, n (%)	3 (20%)	Negative
ANA AC-16, n (%)	1 (6.7%)	
ANA AC-4, n (%)	1 (6.7%)	
ANA AC-8, n (%)	1 (6.7%)	
anti-ENA positivity (RNP, Sm, SSA/B, Scl-70, Jo-1, anti-dsDNA), n (%)	0 (0%)	Negative

AC anti-cell; ANA antinuclear antibodies; ENA extractable nuclear antigens VEGF vascular-endothelial growth factor; VWF Von Willebrand Factor.

**Table 4:** Videocapillaroscopic parameters of the Milan cohort.

	COVID-19 Subjects (n = 15)	Healthy Controls (n = 15)	*p*-Value
Number of fingers with at least one alteration, median (range)	2 (0–8)	1 (0–6)	0.41
Number of capillaries/mm, median (range)	7.8 (4.9–10.0)	8.5 (5.7–10.1)	0.54
Intercapillary distance, μm, median (range)	143.1 (90.7–199.2)	116.9 (31.8–168.9)	0.03
Internal loop distance, μm, median (range)	13.9 (9.3–16.6)	15.4 (12.1–20.2)	0.02
Loop diameter, μm, median (range)	42.0 (29.0–63.6)	47 (32.6–51.6)	0.34
Apical loop distance, μm, median (range)	18.5 (13.3–33.9)	21.4 (12.9–29.1)	0.28
Loop length, μm, median (range)	348.0 (180.8–399.2)	268.5 (190.4–363.9)	0.01
At least one hemosiderin deposit, n (%)	14 (93.3)	4 (26.7)	<0.001
Microhemorrhage, n (%)	14 (93.3)	4 (26.7)	<0.001
Microthrombosis, n (%)	4 (26.7)	0 (0)	0.22
Total hemosiderin deposits per patient, n, median (range)	4 (0–27)	0 (0-1)	<0.001
More than one enlarged loop, n (%)	10 (66.7)	2 (12.5)	0.002
At least one loop with one cross, n (%)	15 (100)	11 (73.3)	0.10
At least one loop with two or more intersections, n (%)	15 (100)	7 (46.7)	0.002
At least one meandering capillary, n (%)	10 (66.7)	1 (6.7)	<0.001
At least one bushy capillary, n (%)	11 (73.3)	1 (6.7)	<0.001

Differences in medians have been evaluated by Mann–Whitney U test and percentage by chi-square test.

**Table 5:** Demographic and clinical characteristics of patients died of COVID-19 of the New Orleans cohort.

AGE (Years)	
Median (range)	62 (47–79)
SEX	
M	7 (47%)
F	8 (53%)
BMI (kg/m^2^)	
Median (range)	36.2 (21.4–62.3)
TIME FROM ONSET TO DEATH (days)	
Median (range)	17 (0–31)
MAIN COMORBIDITIES AND RISK FACTORS	
Arterial Hypertension Diabetes	10 (66%) 7 (47%)
OXYGEN THERAPY	
Total Invasive ventilation	14 (93%) 13 (87%)

## Data Availability

The data presented in this study are available from the corresponding author on reasonable request.
